# Microbial Contamination of the White Coats of Dental Staff in the Clinical Setting

**DOI:** 10.5681/joddd.2009.033

**Published:** 2009-12-15

**Authors:** Harsh Priya, Shashidhar Acharya, Meghashyam Bhat, Mamtha Ballal

**Affiliations:** ^1^ Assistant Professor, Department of Community Dentistry, Manipal College of Dental Sciences, Mangalore, India; ^2^ Head of the Department, Department of Community Dentistry, Manipal College of Dental Sciences, Manipal, India; ^3^ Associate Professor, Department of Community Dentistry, Manipal College of Dental Sciences, Manipal, India; ^4^ Head of the Department, Department of Microbiology, International Center, Kasturba Medical College, Manipal, India

**Keywords:** antibiotic sensitivity, cross infection, dental students, microbial contamination, white coats

## Abstract

**Background and aims:**

Although wearing a white coat is an accepted part of medical and dental practice, it is a potential source of cross-infection. The objective of this study was to determine the level and type of microbial contamination present on the white coats of dental interns, graduate students and faculty in a dental clinic.

**Materials and methods:**

Questionnaire and cross-sectional survey of the bacterial contamination of white coats in two predetermined areas (chest and pocket) on the white coats were done in a rural dental care center. Paired sample t-test and chi-square test were used for Statistical analysis.

**Results:**

60.8% of the participants reported washing their white coats once a week. Grading by the examiner revealed 15.7% dirty white coats. Also, 82.5% of the interns showed bacterial contamination of their white coats compared to 74.7% graduate students and 75% faculty members irrespective of the area examined. However, chest area was consistently a more bacterio-logically contaminated site as compared to the pocket area. Antibiotic sensitivity testing revealed resistant varieties of micro-organisms against Amoxicillin (60%), Erythromycin (42.5%) and Cotrimoxazole (35.2%).

**Conclusion:**

The white coats seem to be a potential source of cross-infection in the dental setting. The bacterial contamina-tion carried by white coats, as demonstrated in this study, supports the ban on white coats from non-clinical areas.

## Introduction

A white coat, apron or laboratory coat (abbreviated lab coat) is a knee-length overcoat or smock worn by professionals in the medical field or by those involved in Laboratory work to protect their street clothes. The garment is made from white cotton or linen to allow it to be washed at high temperature and make it easy to see if it is clean.^1^

There has always been some concern that white coats, nurses' uniforms and other hospital garments, may actually play a part in transmitting pathogenic bacteria in a hospital setting.^2 - 6^ Many articles of clothing and equipment, such as neckties, stethoscopes, pens, lanyards, identify badges along with the doctor’s coat have been noted to carry potential pathogens.^7^Also, dental personnel’s clothing or uniforms (white coat) are spattered by blood, aerosol and saliva and there is a definite risk of infection with various transmissible agents.^8^ There has also been controversy over whether doctors should be barred from wearing white coats in areas such as staff canteens, tea rooms, and libraries. However, wearing a white coat is an accepted part of medical and dental practice. The actual use of white coats and how often they are changed varies greatly among individual doctors and their specialties.

The white coat is associated with medicine, science, and the healing, and it is the most recognized and respected dress of a doctor. Contamination of skin and clothing by “splashes” or touch is practically unavoidable in hospitals. The white coat worn over personnel clothing, is a personal protection equipments (PPE) from such contamination.^9^

There is currently no literature on the contamination of dental personnel’s white coats. Thus, the objective of the present study was to determine the level and type of bacterial contamination present on the white coats of dental staff in order to assess the risk of spread of nosocomial infections by such contact in a dental setting.^9^

## Materials and methods

The present study was conducted in a rural dental care center of Department of Community Dentistry, Manipal College of Dental Sciences, Manipal, India. This center caters to a large rural population and provides free dental care with the help of local voluntary organizations.

A survey of the 51 white coats of dental interns, graduate students and faculty members was done. All the participants wore the white coats as part of the protocol at the dental school. All white coats were full sleeved, made of cotton-polyester material with two pockets at the bottom, one on each side. Also the guidelines of dental school for the students are to launder their own white coats, which they do with varying degrees of regularity.

A pre-tested questionnaire was distributed to the participants assessing the duration of use of their white coats, frequency of washing the white coats and practice of exchanging them. The participants were also asked to grade arbitrarily, their white coat as clean, moderately clean, or dirty. In addition, the cleanness of the coat in appearance was assessed subjectively by the investigator,^10^ as clean, moderately clean, or dirty.

The white coat of each participant was sampled using sterile saline-moistened swabs from the two pre-determined areas, i.e. chest area of the white coat and the pocket mouth, both on the side of the dominant hand.

### Microbiological procedure

After the samples were collected, they were taken to the Department of Microbiology, Kasturba Medical College, Manipal, India, for further analysis. The swabs collected in the study were streaked onto the agar plates which were then incubated overnight at 37°C.^10 , 11^ Examination for total bacterial count and the presence of potentially pathogenic bacteria was done. Suspected colonies were identified by Gram’s method of staining, and antibiotic sensitivities were determined by disc diffusion method according to standard protocol.^11^

### Statistical analysis

Paired sample t-test was performed to compare mean CFU/plate during the procedures. Chi-square test was done to find if any significant difference existed between the participants of the study according to their responses. The cut-off level for statistical significance was taken at 0.05. Data was analyzed using SPSS version 14.

## Results

Table 1 shows the frequency distribution of the participant’s response according to the study variables. Participants of the study were 49% males and 51% females. Majority of the participants washed their white coats once weekly and the practice of exchanging white coats was confirmed by 5.9% of the participants. Table 2 shows the comparison between the participants of the study according to their responses. Majority of the graduate students (73.7%) and faculty members (83.3%) practiced weekly washing regime for their white coats. The habit of exchanging white coats was seen only among interns. The other participants denied the habit. With self-grading, the cleanness of the white coats was regarded as moderately clean by 80% of interns, 73.2% of graduate students and 66.7% of faculty members. With examiner grading of the cleanness of the white coats, however, the percentage of moderately clean white coats decreased. There was a marked difference especially among the interns.

**Table 1:** Frequency distribution of the participants’ response according to the study variables

Variables		n (%)
Participants	Faculty	12 (23.5)
	Graduate Students	19 (37.3)
	Interns	20 (39.2)
Gender	Male	25 (49)
	Female	26 (51)
Frequency of washing white coat	Everyday	4 (7.8)
	Twice a week	13 (25.5)
	Once a week	31 (60.8)
	Once fortnightly	2 (3.9)
	Once a month	1 (2)
Practice of exchanging white coat	Yes	3 (5.9)
	No	48 (94.1)
Self grading white coat cleanliness	Clean	14 (27.5)
	Moderately clean	36 (70.6)
	Dirty	1 (2)
Examiner grading white coat cleanliness	Clean	14 (27.5)
	Moderately clean	29 (56.9)
	Dirty	8 (15.7)
Spills on white coat	Aerosol	39 (76.5)
	Saliva	4 (7.8)
	Others	8 (15.7)

**Table 2:** Comparison between the participants of the study according to their responses

			Subjects		
Variables		Interns n (%)	Graduates n (%)	Faculty n (%)	P value
Frequency of washing white coats	Everyday	3 (15)	1 (5.3)	0	P <0.01
	Twice a week	10 (50)	3 (15.8)	0	
	Once a week	7 (35)	14 (73.7)	10 (83.3)	
	Once fortnightly	0	1 (5.2)	1 (8.3)	
	Once a month	0	0	1 (8.3)	
Exchange of white coats	Yes	3 (15)	0	0	P <0.05
	No	17 (85)	19 (100)	12 (100)	
Self Grading by subjects	Clean	3 (15)	7 (36.8)	4 (33.3)	P =0.36
	Moderately clean	16 (80)	12 (73.2)	8 (66.7)	
	Dirty	1 (20)	0	0	
Examiner Grading of the white coats	Clean	4 (20)	5 (26.3)	5 (41.7)	P <0.05
	Moderately clean	9 (45)	13 (68.4)	7 (58.3)	
	Dirty	7 (35)	1 (5.3)	0	
Spills on the white coats	Aerosol	17 (85)	10 (52.6)	12 (100)	P < 0.001
	Saliva	3 (15)	1 (5.2)	0	
	Others	0	8 (42.2)	0	

Table 3 shows gram positive organisms were the most dominant organisms on the white coats. However, small percentages of gram negative organisms were also found on them. Chest area on the white coat was a more contaminated site compared to the pocket area. Resistant strains against Ampicillin/Amoxicillin, Cotrimoxazole, Erythromycin, Ciprofloxacin/Ofloxacin/Levofloxacin and Gentamicin were found with the antibiotic sensitivity test of the identified bacterial colonies in the study (Table 4).

**Table 3:** Frequency distribution of the white coats showing growth of microorganisms

Organism	Faculty n (%)	Graduates n (%)	Interns n (%)
No growth			
chest area	1 (8.3)	5 (26.3)	3 (15)
pocket area	5 (41.7)	5 (26.3)	4 (20)
Total (%)	6 (25)	10 (26.3)	7 (17.5)
Gram positive organisms			
chest area	9 (75)	13 (68.4)	13 (65)
pocket area	8 (66.7)	12 ( 63.1)	12 (60)
Total (%)	17(70.8)	25 (65.8)	25 (62.5)
Gram negative organisms			
chest area	2 (16.7)	4 (21)	4 (20)
pocket area	1 (8.3)	0 (0)	3 (15)
Total (%)	3(12.5)	4 (10.5)	7 (17.5)

**Table 4:** Frequency distribution of the antibiotic sensitivity of microorganisms

Antibiotics	Total n (%)	Sensitive n (%)	Resistant n (%)
Amoxicillin- Clavulanic acid	2	2 (100)	0
Ampicillin/ Amoxicillin	35	14 (40)	21 (60)
Cefazolin/ Ceftriaxone	5	5 (100)	0
Ciprofloxacin/ Ofloxacin/ Levofloxacin	47	46 (97.8)	1 (2.2)
Cotrimoxazole	37	24 (64.8)	13 (35.2)
Erythromycin	33	19 (57.5)	14 (42.5)
Gentamicin	45	43 (95.5)	2 (4.5)
Piperacillin – Tazobactam	11	11 (100)	0
Penicillin	8	8 (100)	0
Imipenem/ Meropenem	4	4 (100)	0
Cloxacillin	4	4 (100)	0

## Discussion

This study was done to determine the level and type of microbial contamination present on the white coats of the interns, graduate students and faculty members in the dental clinics. Among the two predetermined sites selected for examination of the white coats, chest area showed highest contamination followed by the pocket mouth both on the side of the dominant hand. Another study found the sleeves and the pockets of the white coat as the sites that were most highly contaminated.^9^ As the doctors examine patients, the sleeve of the coat, especially the cuff, is the site that most frequently comes into contact with the patient. Furthermore, transfer of bacteria from sleeves to hands (and vice versa) is also possible. Also, studies have reported that the cuff and the pocket had a significantly higher level of contamination than the back of the white coat.^10 , 12^

Gram positive cocci were isolated from considerably high percentages of the two studied sites on the white coats: 70.8%, 65.8% and 62.5% of the white coats of the faculty, graduate students and interns, respectively. These findings are similar to another study where it has been found that bacteria are most likely to be isolated from the pockets and sleeves of white coats since these were the sites of frequent contact.^13^ The other most common form of microbes found on various sites was Bacillus species. This had not been recorded in previous studies. Gram negative bacilli and other forms of microbes which are considered environmental microorganisms with no clinical significance and skin commensals such as coagulase negative staphylococci were also found in previous studies.^14 , 15^

Because of the high frequency of the patient contact in a busy university clinic, it is reasonable to expect the white coats to become colonized with potentially pathogenic bacteria, which was demonstrated in this study. It has been also seen that the coats become contaminated quickly once worn, as there appears to be little difference between the colony counts according to the frequency of laundering.^9^ In the present study, majority of the graduate students (73.7%) and the faculty (83.3%) washed their white coats once a week. However, 35% interns reported of washing their white coats weekly and 50% washed twice a week. The rate of white coat laundering was better in the present study as compared to the findings of another study,^10^ where 34.4% of students washed their coats once a month; 15.6% once a week and 9.4% twice a month. Remaining 40.6% would wash their coats every two months or even longer. Also another study concluded that most students laundered their coats at either one or four weekly interval with over a third of them laundering it monthly.^9^

A grading of the white coats by the study participants and the examiner was done separately to acknowledge the perception of the white coat’s cleanliness. 80% of the interns, 63.2% of the graduate students and 66.7% of the faculty members considered their white coats as moderately clean whereas the examiner rated 45% of the interns, 68.4% of the graduate students and 58.3% of the faculty members to have moderately clean white coats. This reveals that interns who thought their white coats as clean were not perceived to be clean by the examiner. Hence the students needed to be further trained and a stricter regime of laundering should be followed for the students so that they inculcate the habit.

Antibiotic sensitivity showed resistant species of microorganisms on the white coats against Amoxicillin (60%), Erythromycin (42.5%) and Cotrimoxazole (35.2%). Also, resistance to Gentamycin and Ciprofloxacin was seen in the microorganisms present on two white coats. Multi-drug resistant microorganisms were isolated from white coat and pus samples collected from the patients in medical wards but the antimicrobial sensitivity patterns differed markedly between the two in a previous study.^11^ This suggested the bacteria in the white coats could have been picked up from other sources, especially from the environment — the canteen, college, roads, hostels where the white coats were usually carried.

Therefore, it can be concluded that aprons are potential source of cross infection even in dental setting. Furthermore the bacterial contamination carried by aprons as demonstrated in this study, supports the ban on aprons from non-clinical areas such as canteens and the library and suggests that stricter white coat changing and washing regimes should be implemented.
